# Low determinism in community assembly of transient and resident pelagic fungi across climate zones in Scandinavian lakes

**DOI:** 10.1128/msystems.00691-26

**Published:** 2026-08-20

**Authors:** Pedro M. Martin-Sanchez, Christian Wurzbacher, Laurent Fontaine, Maria Jimenez Lara, Heli Juottonen, Nicolas Valiente, Even Werner, R. Henrik Nilsson, Alexander Eiler

**Affiliations:** 1 Section for Aquatic Biology and Toxicology, Department of Biosciences, University of Oslo 208408, Oslo, Norway; 2 Environmental Microbiology and Cultural Heritage group, Instituto de Recursos Naturales y Agrobiología de Sevilla (IRNAS-CSIC) 83100, Seville, Spain; 3 Chair of Urban Water Systems Engineering, Technical University of Munich 9184, Munich, Germany; 4 Centre for Biogeochemistry in the Anthropocene, Department of Biosciences, University of Oslo 208408, Oslo, Norway; 5 Department of Biological and Environmental Science, University of Jyväskylä 176461, Jyväskylä, Finland; 6 Department of Science and Agroforestry Technology and Genetics, University of Castilla-La Mancha 73073, Albacete, Spain; 7 Department of Biology and Environmental Sciences, Gothenburg Global Biodiversity Centre, University of Gothenburg 3570, Gothenburg, Sweden; University of Illinois Chicago, Chicago, Illinois, USA

**Keywords:** macroecology, community assembly, null models, fungi, eukaryotes, metabarcoding, lakes, freshwaters

## Abstract

In lakes, fungi play pivotal roles in biogeochemical processes, particularly in the decomposition of organic matter. However, these organisms have been underrepresented and taxonomically unresolved in previous research. In this study, we employ high-resolution sequencing to delve into the fungal diversity within two distinct Scandinavian Lake data sets spanning a vast latitudinal gradient across Norway and Sweden, diverse climatic zones ranging from nemoral to arctic, and a wide spectrum of nutrient conditions. Utilizing eukaryotic 18S primers, we reveal that fungi contribute, on average, 2.07% in the Norwegian and 5.4% in the Swedish sequencing data set, with remarkable spikes of up to 55% in forested lakes. Fungus-specific 5.8S-ITS2 sequencing identified the prevalence of several fungal phyla, including *Ascomycota*, *Rozellomycota*, *Basidiomycota*, *Chytridiomycota*, *Aphelidiomycota*, and *Mortierellomycota*. Notably, our research uncovers a striking degree of variability in fungal communities across the studied lakes. Null model analyses on the entire community, as well as subsets of transient and resident members, revealed that deterministic selection plays a secondary role. Instead, community assembly was overwhelmingly governed by stochastic ecological drift across all tested groups. These findings challenge conventional paradigms of eukaryotic communities being strongly guided by deterministic processes and underscore the complexity of community assembly processes across the fungal kingdom. The dominance of ecological drift and stochasticity in the assembly process emphasizes that conservation should target entire communities, processes, and areas instead of single species, as should be the case in deterministically assembled communities.

**IMPORTANCE:**

Fungi perform a range of key functions in freshwater environments. Here, we show that fungi, including members of the phyla *Ascomycota*, *Rozellomycota*, *Basidiomycota*, and *Chytridiomycota*, represent a diverse part of the epilimnetic planktonic community in Scandinavian lakes. Fungal community assembly across northern climate zones is shown to have a low signal for determinism, as environmental sorting was weak in overwriting dispersal-related processes and ecological drift. Furthermore, we expand knowledge of the ecological range of key freshwater fungi and link taxa to biogeographic patterns in lakes.

## INTRODUCTION

Fungi are abundant in many, if not all, aquatic ecosystems, and they have been suggested to play important roles in biogeochemical cycles (1–6). They are key players in organic matter (OM) decomposition from freshwater environments, where fungi can comprise high relative abundances, constituting up to 50% of eukaryotic sequences (7, 8). Nonetheless, our understanding of fungal diversity, quantitative abundance, ecological function, and, in particular, their community assembly and interactions with other microorganisms remains predominantly speculative, unexplored, and absent from contemporary concepts in aquatic ecology and biogeochemistry (3–5, 9, 10). Pioneering efforts have unveiled a high diversity of uncultivated fungal taxa, detected solely through molecular tools (11, 12). They are termed dark fungal taxa, as these lake and river fungi are uncharacterized (13–15).

In contrast to studies on soil ecosystems (16, 17), studies on aquatic fungi are still insufficient or lack adequate spatial and temporal resolution to infer the natural history of freshwater fungi. An initial global survey compiled from the literature has provided valuable insights into the ecology and biogeography of aquatic hyphomycetes (18). Nonetheless, unraveling the ecology and biogeography of diverse fungal groups requires an extensive array of such studies, with sufficient environmental metadata, to shed light on dark fungal taxa and their roles in aquatic ecosystems. Moreover, climate change and other anthropogenic stressors are currently shifting the distribution and abundance of fungal species (19), emphasizing the need to understand the mechanisms governing community assembly and the regulation of fungal diversity for conservation and other management actions to be successful. Previous studies on aquatic fungi have shown contrasting results, emphasizing local environmental conditions (20, 21) or ecological drift (22) as the main drivers. While prokaryotic community assembly is predominantly deterministic and largely driven by environmental sorting (23, 24), this may differ significantly in freshwater fungi, where stochastic processes, dispersal limitation, biotic interactions, or historical processes may exert greater influence over community assembly.

Distinguishing between deterministic selection and stochastic drift in fungi is important to predict cross-kingdom symbioses and host-pathogen dynamics in aquatic environments (25). If community assembly is dominated by ecological drift, as suggested by a recent freshwater meta-analysis (26), then functional recovery following disturbance becomes inherently less predictable. This shift toward stochasticity makes species-specific conservation considerably more difficult than in systems governed by deterministic niche filtering.

To shed light on the ecological phenomena of community assembly, a statistical toolbox is used, consisting of several complementary approaches that all have their strengths and limitations. Recently, null model approaches that facilitate quantitative comparisons of assembly processes have been increasingly used (27, 28). Among them, methods to discern the elements of metacommunity structure (EMS) have sought to distinguish between communities randomly assembled from those shaped by species-sorting processes (29, 30). Co-occurrence indices (31) and incidence-based (Raup-Crick) beta-diversity (β_RC_) (32) have been used to parse deterministic and stochastic assembly processes (33, 34). Additionally, the integration of phylogenetic information into null model approaches, based on the premise that phylogenetic relatedness mirrors shared environmental response traits (35), has expanded the toolkit for disentangling community assembly (36). Stegen et al. (28, 37) combined null model approaches based on phylogenetic and abundance-based (Raup-Crick) beta-diversity (β_RCbray_) measures. This approach enables the quantitative estimation of the relative importance of processes such as selection, drift, dispersal limitation, and mass effects, while also distinguishing between heterogeneous (i.e., beta-diversity enhancing) and homogeneous (i.e., beta-diversity diminishing) selection processes.

In the present study, we use high-throughput amplicon sequencing of (i) a universal region of the small subunit (SSU) 18S rRNA gene (V6-V8) spanning all eukaryotic diversity and (ii) other regions of the rRNA operon including the 5.8S gene and the internal transcribed spacer 2 (ITS2), which are recognized as a universal fungal barcode (16, 38). Our main goal is to target fungal diversity within two independent investigations of lakes across Scandinavia (Norway and Sweden), covering a gradient from nemoral to arctic zones. Through statistical analyses, we aim to identify the environmental factors most strongly related to fungal diversity, including catchment land use/cover, hydrological properties, and geochemical parameters. We seek to disentangle the roles of stochastic (i.e., ecological drift) and deterministic (i.e., environmental sorting and dispersal) factors in shaping the assembly of fungal communities across lake ecosystems.

## RESULTS AND DISCUSSION

### Characteristics of surveyed Norwegian and Swedish lakes

We set out to investigate and quantify the diversity of epilimnic freshwater fungi across a broad range of latitudes, spanning from 55.4° to 68.3° (Fig. S1). This represents the latitudes with the highest concentration, area, and perimeter of inland water bodies (33) globally. The sampled Norwegian and Swedish lakes presented a diverse array of ecological contexts, covering vegetation zones from nemoral to arctic (subalpine). Metadata associated with each of the 160 sampled lakes, or at least a substantial subset thereof (61 Norwegian and 83 Swedish lakes where sufficient fungal sequences were retrieved), included latitude, longitude, temperature, chlorophyll concentration, nutrients, and catchment properties. Summary statistics of metadata are shown in the Table S1.

Beyond the distinction in latitude, catchment properties, and temperature (range 0.2°C–24.7°C), the sampled lakes varied in nutrient content. Total organic carbon (TOC) in the lakes ranged from 0.6 to 116 mg L^−1^ (with a median of 13.3 mg L^−1^ in Norwegian and 9.4 mg L^−1^ in Swedish lakes), total phosphorus (TP) from 2 to 136 µg L^−1^ (median of 8.9 µg L^−1^ in Norwegian and 10 µg L^−1^ in Swedish lakes), and total nitrogen (TN) from 50 to 1,280 µg L^−1^ (median of 271 µg L^−1^ in Norwegian and 360 µg L^−1^ in Swedish lakes). Additionally, we recorded pH levels ranging from 4.4 to 9.0 (median of 6.5 in Norwegian and 6.7 in Swedish lakes). In both data sets, TOC, TP, and TN correlated positively with each other and with turbidity, and negatively with latitude (Fig. S2). The Norwegian lakes varied in size from 0.08 to 14.5 km^2^ with a median of 0.98 km^2^. In contrast, the Swedish lakes ranged from 0.03 to 14 km^2^ with a median of 0.44 km^2^ (Table S1).

### Fungal contribution and alpha diversity in freshwater lakes

Utilizing metabarcoding of the eukaryotic SSU rRNA gene, our study revealed that fungal taxa constituted a substantial portion of the overall diversity within some of the studied lakes, spanning a wide range from 0.18% to as much as 55% (Fig. S3). Specifically, the Norwegian data set displayed an average of 2.07% fungal reads, while the Swedish data set exhibited an average of 5.41% fungal reads. These observations align with previously reported ranges for fungal relative read abundances in lakes (7, 14, 39) and ponds (40). These findings are also consistent with studies involving measurements of phospholipid-derived fatty acids in seston biomass of numerous lakes and kettle holes in northeast Germany, where fungi typically accounted for an average of 9.2% ± 5.2% of the biomass in the pelagic zone (41).

**Fig 1:**
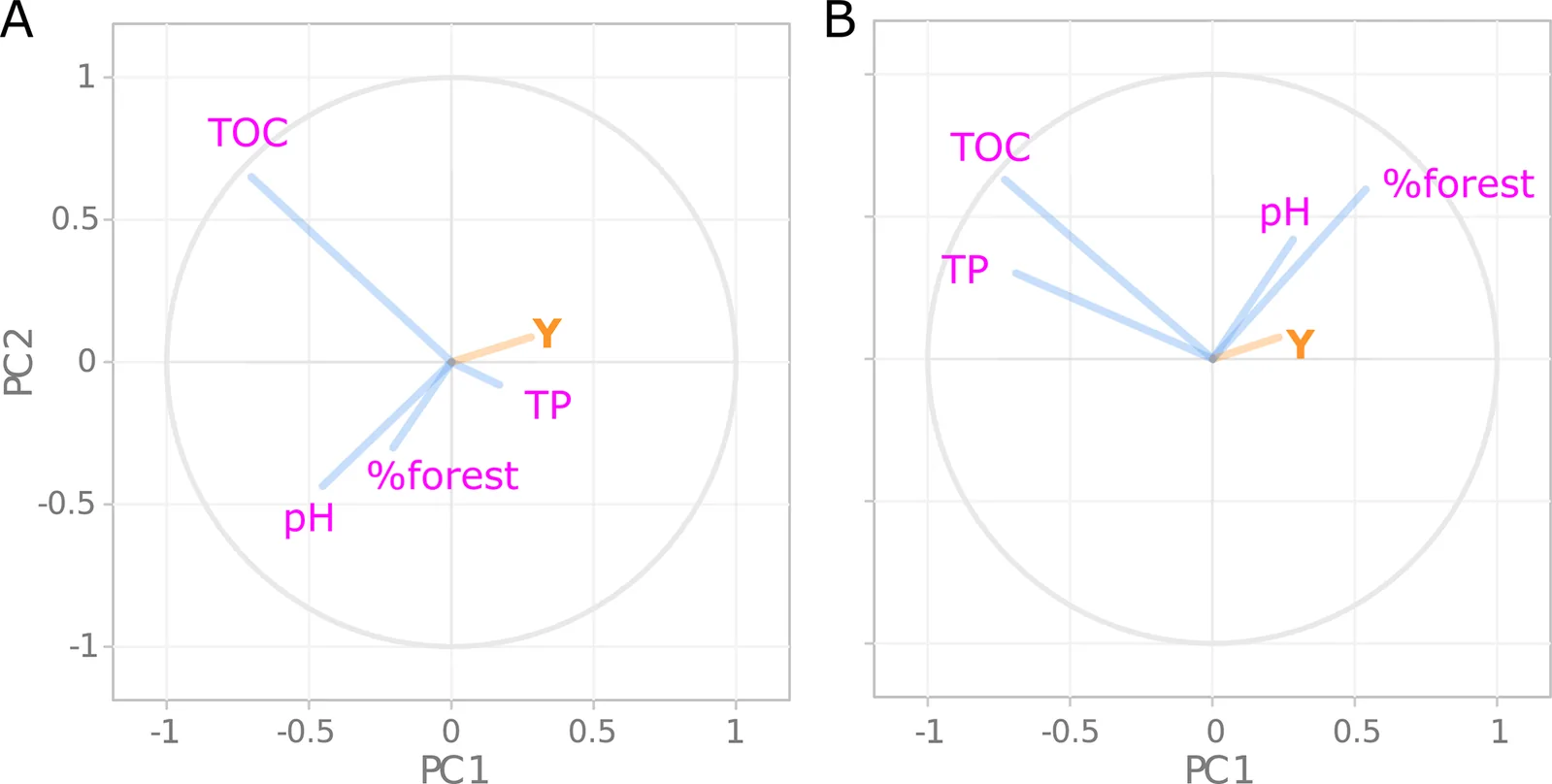
Plot representing the results from a PLS regression model that predicts fungal contribution (in orange) to the eukaryotic community from environmental data (in blue). Environmental variable arrows with the smallest angle (i.e., closest) to the fungal arrow (“Y”) have the highest correlation with fungal contribution. The models are based on data from 73 Norwegian (**A**) and 83 Swedish (**B**) lakes.

Our analysis, employing partial least squares (PLS) modeling, revealed that the relative abundance of fungal SSU sequences in relation to those from other eukaryotes increased with catchment land cover classified as forest and decreasing pH values in the Swedish data set while in the Norwegian data set, these patterns were not clear (Fig. 1). Latitude used as a proxy for climate did not reveal any significant relationships. It is of note that the highest fungal relative abundances (over 20%) were found in dystrophic (high proportion of forests in the catchment) lakes. Dystrophic lakes are often characterized by anoxic bottom waters (hypolimnion) and high inputs of terrestrial organic matter, giving them their distinctively dark, tea-colored appearance. This reflects that fungi, with their larger genomes and higher enzymatic capabilities, can thrive best in lakes with a higher content of recalcitrant organic matter, which is analogous to the reported situation for ectomycorrhizal fungi in terrestrial environments (42). The resulting low light conditions and nutrient limitations, especially when it comes to nitrogen and phosphorus, restrict primary productivity in dystrophic lakes, tipping the balance towards heterotrophic eukaryotes such as fungi.

The fungus-specific sequencing libraries, covering the partial 5.8S gene and ITS2 region, yielded 631 and 1,271 fungal operational taxonomic units (OTUs) in the Norwegian (comprising 51 lakes) and Swedish (comprising 84 lakes) data sets, respectively. After rarifying to 700 reads due to uneven read coverage per sample, observed OTU richness (ranging from 3 to 79) and evenness (Pielou’s evenness ranging from 0.02 to 0.94) varied widely across the data sets but could not be explained by any of the environmental properties as tested by correlation and PLS analysis. In the fungus-specific sequencing libraries, percentages of OTUs assigned to the kingdom *Fungi* varied from 44% for the Norwegian lakes to 59% for the Swedish ones, which is in line with previous results from surface waters in Iceland, where 54% of the reads were recovered as fungi (43). A high proportion of OTUs were not assigned at the kingdom level (33% and 21% for Norwegian and Swedish data sets, respectively) or matched non-fungal organisms (23% and 20%), mainly members of the kingdoms *Alveolata* (phylum *Ciliophora*) and *Viridiplantae*, reflecting the relatively low specificity of the selected fungal primers (ITS3-Mix2 and ITS4-cwmix1/2). Similar results were obtained in a previous fungal DNA metabarcoding study, using the primers fITS7a and ITS4 to amplify the ITS2 region of Scandinavian lakes (14). Comparing it to the eukaryotic data (SSU libraries), both *Alveolata* (*Ciliophora*) and *Viridiplantae* (*Chlorophyta*) were found to be prevalent and abundant (Fig. S3).

A large portion of the fungal OTUs was unique to specific lakes, with 72% (438) of Norwegian and 71% (549) of the Swedish OTUs occurring in only a single lake. An additional 19% were observed in two or three lakes within the Norwegian and Swedish systems. The remaining 9.7% of Norwegian (51) OTUs and 10.0% of Swedish (93) OTUs were distributed across more than three lakes. These common OTUs often dominated the fungal community, with a combined relative abundance ranging from 0.3% to 99% per Norwegian lake and 0% to 100% per Swedish lake, averaging 54% and 71% across the Norwegian and Swedish data sets, respectively (Fig. S4).

In the Norwegian data set, the most prevalent OTU, identified as *Hyaloscyphaceae* (*Ascomycota*), was found in 20 lakes, while the dominant OTU in the Swedish data set was an unassigned *Rozellomycota* (previously *Cryptomycota*) occurring in 49 lakes. The presence of numerous OTUs restricted to specific lakes, along with the low prevalence among most OTUs (see Fig. S5), suggests a high level of among-lake richness. This was further substantiated by species accumulation curves (SAC) (see Fig. S6), indicating that the OTU pool remains undersampled across the broad environmental gradients sampled, despite the sequencing efforts that detected 631 and 1,271 OTUs in the rarefied Norwegian and Swedish data sets, respectively. Most likely, the limited specificity of ITS primers discussed above has decreased the effective sequencing depth for recovering the full fungal diversity of the studied samples. In addition, it is important to note that these results, based on denoised data designed to remove artificial diversity introduced during sequencing, may still contain incorrect sequences, potentially inflating richness and distorting community composition (44, 45).

### Deterministic versus stochastic fungal community assembly

A first visual inspection of beta diversity using non-metric multidimensional scaling (NMDS) did not reveal any clear latitudinal pattern for either data set (Fig. S7). Distance-based redundancy analysis (dbRDA) and fitting environmental variables to the ordinations, as well as partial Mantel tests, suggested that total fungal diversity was only partly driven by the measured environmental variables (Table S2). Next, we explored whether there was a difference between transient and resident fungi. To isolate the assembly mechanisms of true aquatic fungi from the terrestrial spore rain, we partitioned the community into resident and transient subsets using an operational taxonomic framework. We defined the early-diverging *Chytridiomycota* and *Rozellomycota* as resident “indwellers” due to their evolutionary adaptations for aquatic life (e.g., motile zoospores) and their obligate integration into pelagic food webs as parasites and saprotrophs (2). Conversely, *Ascomycota* and *Basidiomycota* were operationally defined as transient “immigrants.” While we acknowledge that certain *Dikarya* taxa have secondarily adapted to freshwater, at the phylum level across forested catchments, their presence in the pelagic zone is overwhelmingly driven by allochthonous inputs of wind-dispersed spores and terrestrial runoff. This operational split allows for a conservative test of whether low determinism is a universal feature of lake fungi or an artifact due to the dispersal of terrestrial-derived fungi.

RDAs using environmental data explained between 3% and 30% of the variability of the community composition, with the lowest explanatory power for the transient community in the Swedish data set (Table S3). Partial Mantel tests were inconclusive since, in the Norwegian data set, geographic distance and in the Swedish data set environmental properties explained around 20% of the variability, and also resident and transient community members behaved in various ways (see Table S3 for details). These results indicate that, beyond profound differences in explanatory variables, deterministic processes can partially overwrite random ecological drift (stochastic processes) in assembling lake fungal communities across climate gradients.

**Fig 2:**
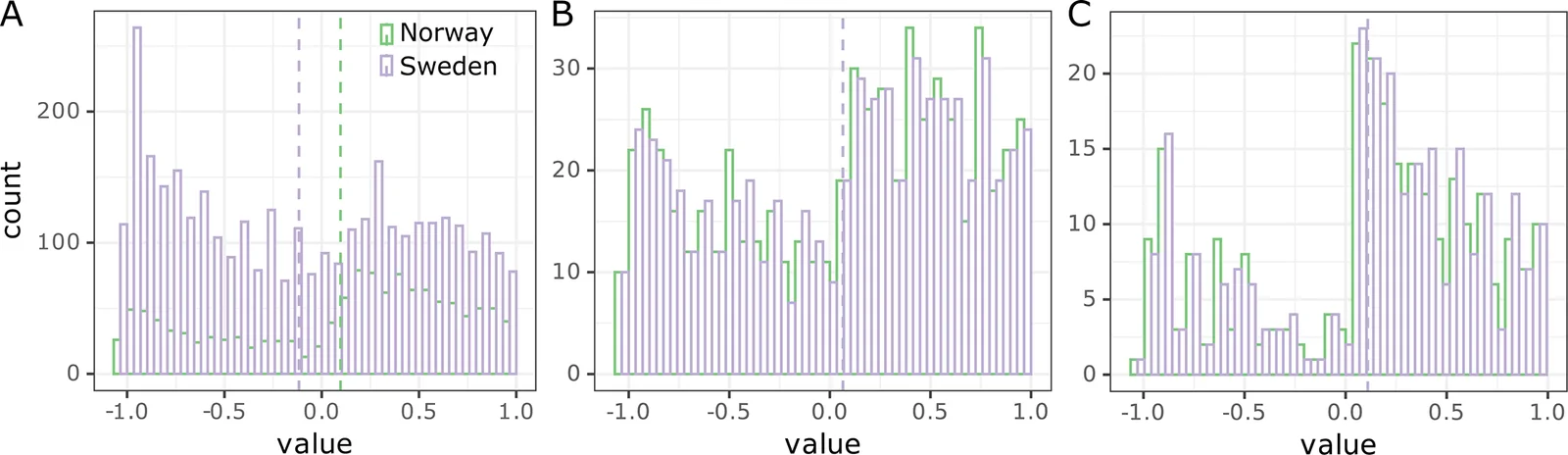
Variation of incidence-based (Raup-Crick) beta-diversity (β_RC_) in the total fungal communities from the Norwegian (green; 61 lakes) and Swedish (blue; 83 lakes) data sets (**A**). In panel** B**, the variation of β_RC_ in the operationally defined transient (*Ascomycota* and *Basidomycota*; samples from 35 Norwegian and 66 Swedish lakes) is given; in panel **C**, the variation of β_RC_ in the resident lake fungi (*Chytridiomycota* and *Rozellomycota*; samples from 23 Norwegian and 56 Swedish lakes) is given. Values close to −1 suggest deterministic processes favoring similar communities, and those close to +1 favor dissimilar communities. The dashed line refers to the mean values of the respective lake data set.

To dissect the interplay between stochastic and deterministic processes shaping fungal communities over the sampled climate gradient, we conducted Monte Carlo simulations to analyze incidence-based co-occurrence patterns (46) and Raup-Crick-based beta-diversity (β_RC_) (32) of the OTUs within the study lakes. Our findings revealed standard effective sizes that did not consistently significantly (*P* > 0.05) deviate from random within the Swedish and Norwegian data sets across the co-occurrence statistics such as the C-score—cscore, count of checkerboard species pairs*—*checker, and the number of species combinations*—*combo, when employing different randomization approaches considering fixed, equiprobable, or proportional constraints for rows and columns (Table 1). In both data sets, signals of determinism in co-occurrence patterns were observed (Table 1); however, β_RC_ values <−0.95 and >0.95 were scarcely present (Fig. 2). Consequently, these findings suggest that ecological drift plays a pivotal role in the assembly of fungal communities (31) across Scandinavia while deterministic processes like environmental sorting and species interactions (31, 47), as well as biogeographic and evolutionary history (48, 49), are not important over space and time (24).

**TABLE 1:** Results from incidence-based EcoSimR (41)^*a*^

Simulation	Rows	Columns	Checker	C-score	Combo
Norwegian					
SIM2	Fixed	Equiprobable	−0.39/0.41	13.1/>0.001	−3.24/0.002
SIM3	Equiprobable	Fixed	0.02/0.97	NA^*b*^	−10.9/>0.001
SIM9	Fixed	Fixed	−0.72/0.58	0.88/0.19	−1.58/0.12
Swedish					
SIM2	Fixed	Equiprobable	1.75/0.032	97.7/>0.001	−6.18/>0.001
SIM3	Equiprobable	Fixed	−0.03/1	NA	−10.4/>0.001
SIM9	Fixed	Fixed	ΝΑ/1	2.45/>0.001	0.26/0.51

^*a*^ Utilizing Monte Carlo randomization *z*-scores for various co-occurrence indices, including C-score (50), the number of checkerboard species pairs (42) (Checker), and the number of species combinations (23) (Combo), were estimated for the Norwegian and Swedish fungal lake amplicon variants (OTUs). Fixed rows-fixed column and fixed rows-equiprobable column simulations were utilized to minimize type I and type II errors (25).

^*b*^ NA, not assessed.

**Fig 3:**
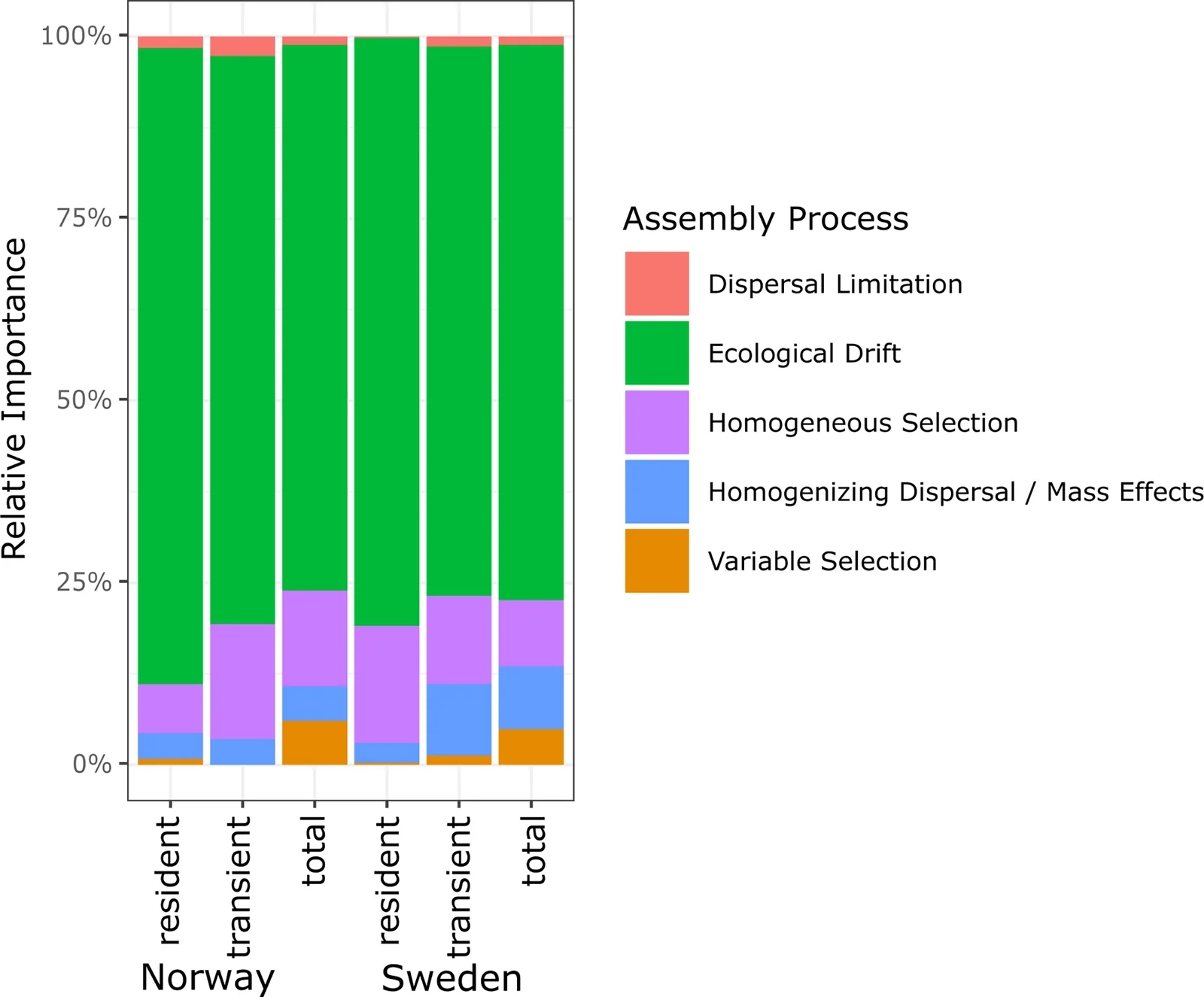
Relative importance of different community assembly processes expressed as the percentage of community pairs. Processes included dispersal limitation (|β_NTI_| < 2, and β_RCbray_ > 0.95), ecological drift (|β_NTI_| < 2, and |β_RCbray_| < 0.95), homogenous selection (β_NTI_ < −2), homogenizing dispersal or mass effects (|β_NTI_| < 2, and β_RCbray_ < −0.95), and variable selection (β_NTI_ > 2). The relative importance is given for the total fungal communities (total), the operationally defined resident lake fungi (*Chytridiomycota* and *Rozellomycota*; resident), and the transient terrestrial fungi (*Ascomycota* and *Basidiomycota*; transient).

To further resolve and estimate the relative importance of deterministic assembly processes, we applied the quantitative process estimate (QPE) framework (24). The results displayed similar general patterns in the two datasets (Fig. 3). In both the complete Norwegian and Swedish data sets, ecological drift was the dominant assembly process based on pairwise comparisons (|β_NTI_| < 2, and |β_RCbray_| < 0.95; 74.9 and 76.2% of pairwise comparisons in the Norwegian and Swedish data sets, respectively). Homogeneous (β_NTI_ < −2) selection accounted for 13.2% in Norway and 9.1% in Sweden, while variable (β_NTI_ > 2) selection accounted for 6.0% and 4.9%, respectively. Homogenizing dispersal or mass effects (|β_NTI_| < 2, and β_RCbray_ < −0.95) accounted for 4.7% and 8.6%, and dispersal limitation or historical contingency (|β_NTI_| < 2, and β_RCbray_ > 0.95) accounted for 1.2% in both data sets (Fig. 3). The low selection confirms the low power of environmental factors in predicting fungal assembly (envfit results and RDA).

The dominance of stochastic processes (ecological drift) in lake fungi community assembly can potentially be explained by the transient nature of many identified taxa; i.e., allochthonous spores and hyphae from the surrounding catchment. Hence, we partitioned the data to compare transient (terrestrial wash-in) and resident (true aquatic) fungal communities. When evaluating these groups independently, we found that assembly mechanisms remained remarkably consistent. Ecological drift dominated in both groups: the transient *Ascomycota* and *Basidiomycota* (78.0% in Norway and 75.4% in Sweden), and the resident *Chytridiomycota* and *Rozellomycota* (87.4% in Norway and 80.7% in Sweden). Interestingly, homogeneous selection was the primary deterministic force structuring these specific subsets, accounting for 15.8% of assembly in Norwegian transient fungi and 16.1% in Swedish resident fungi (Fig. 3).

By applying the “indweller-immigrant” framework (2) to our data sets, we directly tested whether “true” aquatic fungi assemble differently than terrestrial transient ones. Our findings confirm a global synthesis regarding the strength of environmental filtering (26). Like Sanyal et al. (26), we demonstrate that factors such as TOC and pH (Fig. 1) impose some environmental sorting. However, our QPE demonstrated that deterministic selection (primarily homogeneous) provides only a weak structural backdrop for aquatic mycobiomes across the Scandinavian lake gradient. We found that ecological drift overwhelmingly overwrote most of the selective pressures, governing the assembly of both “true” aquatic indwellers (*Chytridiomycota* and *Rozellomycota*) and terrestrial immigrants (*Ascomycota* and *Basidiomycota*). Yet, homogeneous selection emerged as the primary deterministic factor, though its impact varied between the data sets and ecological subsets. For transient terrestrial fungi (*Ascomycota* and *Basidiomycota*), homogeneous selection peaked in the Norwegian data set (15.8%). In this context, selection favors terrestrial clades possessing traits that allow them to persist in the water column, while broadly filtering out less persistent members of the terrestrial spore rain. Conversely, for true aquatic residents (*Chytridiomycota* and *Rozellomycota*), homogeneous selection was highest in Sweden (16.1%). As obligate aquatic parasites and saprotrophs, these lineages are highly adapted to the relatively uniform physical constraints of the pelagic zone, which selects for a conserved set of traits across the latitudinal gradient.

The lack of a universal trend differentiating residents from transients across both regions underscores our primary conclusion. When deterministic selection is weak, it fails to establish a dominant, repeatable macroecological pattern. Rather than being governed by strict environmental filtering, the assembly of both transient and resident lake fungi is overwhelmingly dictated by neutral processes, local historical contingencies, and random demographic chance. This dominance of ecological drift suggests that within these specific boreal and subarctic networks, local environmental gradients simply do not exert enough selective force to overcome the profound stochasticity of fungal population dynamics and random dispersal events.

### Taxonomic distribution of fungi across Scandinavian freshwater lakes

The most abundant fungal phyla across the Norwegian lakes were *Ascomycota* (50% of the reads), *Rozellomycota* (19%), *Basidiomycota* (5.3%), *Chytridiomycota* (2.1%), *Aphelidiomycota* (1.9%), and *Glomeromycota* (1.7%). In Swedish lakes, the dominant phyla were *Rozellomycota* (29%), *Ascomycota* (28%), *Basidiomycota* (21%), *Chytridiomycota* (16.5%), and *Mortierellomycota* (0.2%).

The majority of reads in both data sets were not assigned at the genus level, with percentages of unidentified reads per sample ranging from 3% to 100% (Fig. S8). The low assignment percentage can be partially due to the conservative method used for the taxonomic assignment by the lowest common ancestor (LCA) analysis, as well as the poor representation of the aquatic (freshwater and marine) fungi in the UNITE reference database. Previous studies have identified aquatic ecosystems as priority targets for the recovery of novel lineages within the early diverging fungi affiliated to phyla such as *Rozellomycota*, *Chytridiomycota*, and *Aphelidiomycota* (2), which were abundant in our survey, and especially in the Swedish data set.

Regarding the limited fraction of OTUs identified at the genus level, the most common genera in the Norwegian data set were taxa associated with plants (both saprobic and pathogenic fungi such as *Ascocoryne, Ramularia, Naevala,* and *Oidiodendron*) and lichens (*Epithamnolia*). *Lemonniera*, a known aquatic genus in *Leotiomycetes* (51), was also abundant in some samples. In contrast, the most abundant genera in the Swedish data set were yeasts belonging to the basidiomycete orders *Tremellales* (*Naganishia, Vishniacozyma,* and *Filobasidium*, which were formerly referred to the genus *Cryptococcus*) and *Sporidiobolales* (*Rhodotorula*), and the ascomycete order *Dothideales* (*Aureobasidium*). Previous studies have reported the genera *Cryptococcus*, *Rhodotorula,* and *Aureobasidium* in freshwater systems (2, 6, 14, 52). In addition, the ubiquitous plant pathogen genus *Alternaria* was also abundant in the Swedish data set.

The higher number of genera (and OTU richness) identified in the Swedish data set (284 genera) compared to the Norwegian data set (159 genera) is most likely due to the wider latitudinal range sampled. From the total of 355 genera identified after combining both data sets, 25% (89 genera) of these were shared among the Norwegian and Swedish data sets (Fig. S9). Some of these overlapping genera have been identified in previous lake studies with diverse ecological characteristics: *Aureobasidium* (ubiquitous and widespread)*, Candida* (plant- and food-associated), *Chytridium* (aquatic pathogens), *Rhodotorula* (ubiquitous and widespread), *Trechispora* (wood-associated)*,* and *Rozella* (endoparasitic) (14, 40, 53).

Our results on the fungal taxonomic composition of Scandinavian lakes support previous studies (14, 54), which reported that fungal communities of freshwater systems contain resident taxa, those active members of the community with a clear aquatic origin, as well as transient taxa, with a terrestrial origin, whose fungal structures have been washed into aquatic habitats (55). Furthermore, in this comparison of Swedish and Norwegian data, the seasonality has to be taken into account. The Norwegian water samples were collected when the autumn leaf fall took place, and thus fungi linked to plant material degradation were prominently detected, while the August samples from the Swedish lakes instead reflected planktonic fungi that are linked to the autotrophic food-web, like yeasts and parasites (*Rozellomycota*).

**Fig 4:**
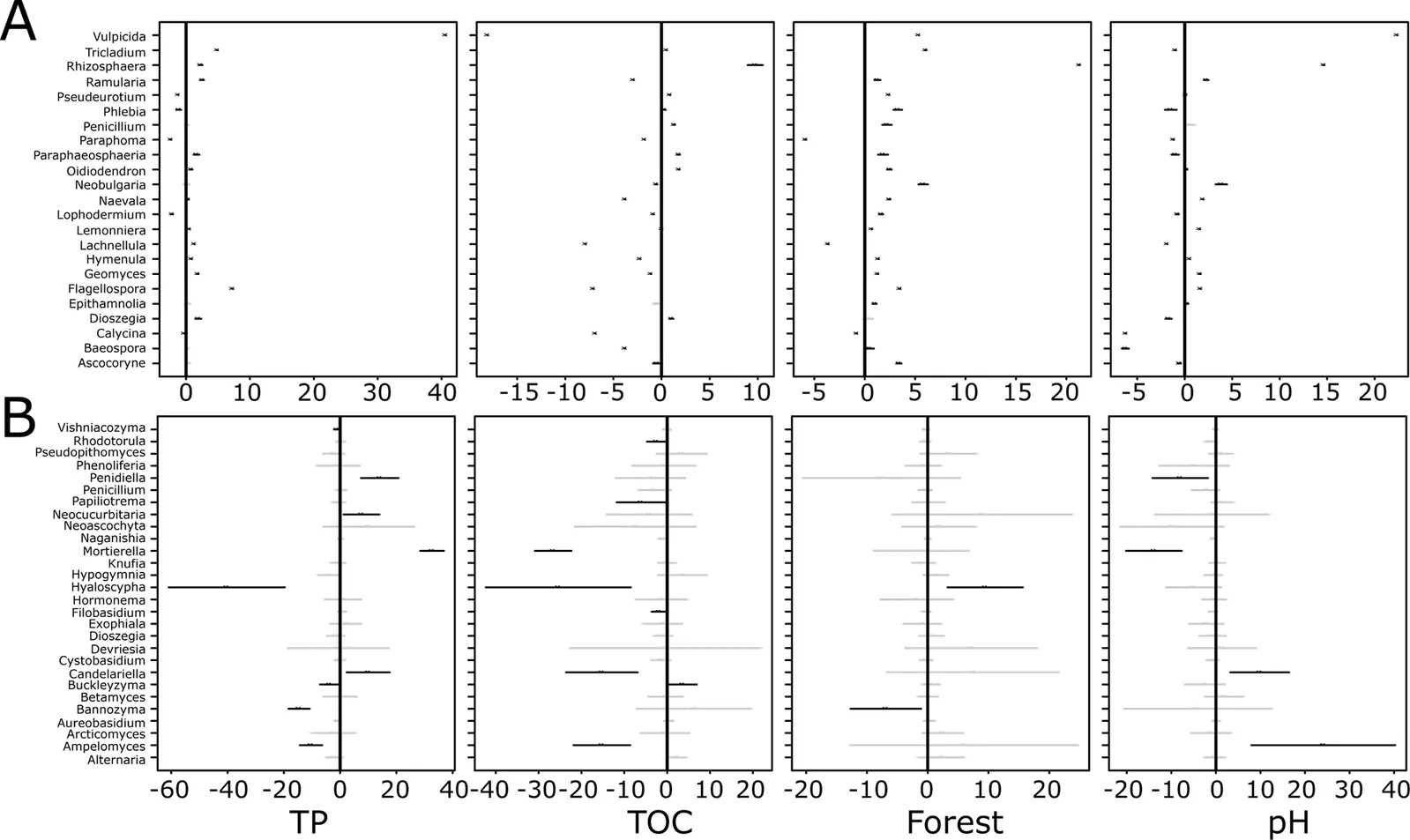
Results from a general linear latent model (gllvm) on fungal genera (with at least 1,000 reads) from Norwegian (**A**) and Swedish (**B**) lakes. The estimated coefficients for predictors and their confidence intervals (TP, total phosphorus; TOC, total organic carbon; Forest, forest coverage in the catchment; and pH) allow studying the nature of effects of environmental variables on fungal genera. Point estimates (ticks) for coefficients of the environmental variables and their 95% confidence intervals (lines) are given, with those colored in black denoting intervals not containing zero. Evaluation statistics of the models are shown in Fig. S10 and S11.

Redundancy analysis indicated that the measured environmental lake properties could only explain a minor fraction of the taxonomic shifts at the phylum and genus levels (Table S2). Using generalized linear latent models (gllvms) on individual genera revealed some strong relationships with lake properties such as nutrient status (i.e., total phosphorus) and total organic carbon concentrations, as well as landscape features (% of forest in the catchment) (Fig. 4; Fig. S10 and S11). For example, *Anguillospora*, a genus previously described to be common in freshwaters, was predicted to increase in contribution in lakes with high nutrient content while being less common in forested lakes with high TOC concentrations. *Lemmoniera* is another common freshwater fungal genus that was positively correlated with nutrient status (i.e., TP), while no relationships with the other lake properties could be identified. As such, the gllvms expand the knowledge about the ecological range of key freshwater fungal genera and provide insights into the ecological drivers of individual genera. This information is essential for species distribution modeling to better comprehend the magnitude of fungal biodiversity change, reliably anticipate these changes, and inform conservation actions (56).

### Conclusion

Our observations challenge the predictions of the size-dispersal hypothesis, which posits that small organisms, such as most fungi, are primarily structured deterministically due to their susceptibility to species sorting and remarkable capacity for dispersal (57–59). However, the excessive dispersal (homogenizing dispersal; β_NTI_< 2, and β_RCbray_ < −0.95) that transfers substantial numbers of organisms across ecosystem boundaries (commonly referred to as “mass effects”) attenuates the impact of local environmental conditions (60), potentially distorting selection processes, as reflected in most pairwise comparisons having a |β_NTI_| < 2. Furthermore, variations in species sorting mechanisms, as indicated by the results from the gllvm, across fungal taxa can introduce additional stochasticity, as well as the variability in dispersal mechanisms among different fungal taxa (61, 62), contributing to the absence of a universal dispersal process across the surveyed landscapes (62). Not determined, recent diversification events and any historical impediments to gene flow (63) can also contribute to stochasticity, thus presenting a major challenge in untangling the intricacies of community assembly processes (64–66). This stochasticity dictates the assembly of both transient and operationally defined resident aquatic fungi alike, presenting a major challenge for modeling alpha and beta diversity dynamics across climate zones.

The overwhelming dominance of ecological drift carries profound implications for aquatic biotic interactions, particularly regarding pathogenesis and symbiosis. In deterministically assembled communities, the distribution of fungal pathogens or symbionts can often be predicted by modeling environmental proxies, such as temperature or nutrient gradients. However, when community structure is dictated by random dispersal and demographic chance, environmental filtering loses its predictive power. Consequently, encounter rates between resident aquatic fungi, such as parasitic *Chytridiomycota* or *Rozellomycota*, and their potential planktonic hosts are thus suggested to be governed by erratic spatial overlaps rather than shared environmental optima. This suggests that strict, highly specialized cross-kingdom symbioses may be rare in these networks, favoring opportunistic or generalist interaction strategies instead. Furthermore, the decoupling of pathogen presence from local water chemistry means that traditional, environmentally based early warning systems for monitoring fungal pathogen dispersal will likely fail. Anticipating outbreaks will instead require a shift toward high-frequency, direct molecular monitoring of the taxa themselves.

Ultimately, recognizing the stochastic nature of these assemblages must guide future management and conservation efforts. In highly deterministic communities, justified conservation plans typically recommend the protection of keystone species and specific niches (67, 68). In contrast, for fungal communities driven by strong ecological drift, conservation frameworks must focus on protecting overall communities, underlying ecological processes, and entire catchment areas instead of single species (69).

## MATERIALS AND METHODS

### Sampling Norwegian lakes and measurements of environmental state variables

In the autumn (October–November) of 2019, we gathered surface water samples from 73 lakes in southeastern Norway (Fig. S1). These lakes were specifically selected as a subset of those monitored in a comprehensive 1,000-lake survey across Norway, conducted by the Norwegian Institute of Water Research (NIVA). This synoptic survey aimed to replicate earlier campaigns carried out in 1986 and 1995 (70), and covered a broad spectrum of water quality properties, including dissolved organic matter (DOM) and nutrients (71), as well as catchment properties and land use (72). Sampling took place in late autumn, coinciding with or following lake overturn. Samples were collected approximately 4 m from each lake’s shoreline using a sampling rod equipped with a sampling beaker. Whenever feasible, we utilized the lake’s outlet as the sampling point. A composite water sample of approximately 2 L was collected in a sampling bucket with minimal physical disturbance. For DNA analyses, water samples (from 0.2 to 1.2 L) were filtered through 0.2-μm pore size Sterivex cartridges (Millipore) in duplicate, using sterile syringes. These filters were then immediately frozen in liquid nitrogen on-site until further processing. Upon sample collection, we immediately measured water temperature (T), pH, and electrical conductivity (EC). Additionally, 50 mL of unfiltered water was collected from each site and stored at 10°C in polypropylene tubes. Upon arrival at the laboratory, these subsamples were frozen at −20°C until analysis for TOC, TN, and TP at the University of Oslo, Norway. TOC was determined by infrared CO_2_ detection following catalytic high-temperature combustion using a Shimadzu TOC-VWP analyzer. TN was quantified by detecting nitrogen monoxide through chemiluminescence with a TNM-1 unit attached to the Shimadzu TOC-VWP analyzer. TP was measured as phosphate after wet oxidation with peroxodisulfate using an auto-analyzer.

### Sampling of Swedish lakes and measurements of environmental state variables

In August 2014, as part of a comprehensive national lake monitoring program (for more details see reference 23), we obtained samples from 119 Swedish lakes. Water samples for chemical analysis were acquired from a depth of 0.5 m using a 0.5-m-long tube sampler of Ruttner-type design. For microbiological analysis, samples were collected from the entire epilimnion, typically extending between 2 and 8 m in depth, employing a 2-m-long tube sampler. These epilimnion samples were combined in a large container, and subsequently, subsamples of 100 and 500 mL were filtered using a peristaltic pump through 142 mm Millipore polycarbonate filters with a 0.2-μm pore size, continuing until the filters became clogged. The filters were promptly stored at −80°C until further processing.

Geographical data, including lake area and catchment area, were sourced from the Svenskt Vattenarkiv database (Swedish Water Archive, SMHI, 2012). Information on catchment land use and cover, such as the percentages of forest, agriculture, and urban areas, was obtained from the Swedish Land Cover Database (Naturvårdsverket, 2014), which is a subset of the CORINE Land Cover data (European Environment Agency, 2014).

Water physicochemical data at the time of sampling, collected as part of the Swedish national lake monitoring program, are publicly accessible through the national data host (https://www.slu.se/en/departments/aquatic-sciences-assessment/data-host/). These physicochemical measurements adhered to international (ISO) or European (EN) standards.

### DNA extraction and eukaryotic sequencing of Norwegian lakes

Total DNA was extracted from the Sterivex cartridges using the DNeasy PowerWater Sterivex Kit (Qiagen, Germany). Eukaryotic SSU rRNA gene amplicons were sequenced following established protocols (73). In short, the eukaryotic primers TAReuk454FWD1 5′-CCAGCASCYGCGGTAATTCC-3′ and V4 18S Next.Rev 5′-ACTTTCGTTCTTGATYRATGA-3′ (74) were utilized, equipped with Nextera adapters and indices. The pooled samples were subsequently sequenced at the IMR sequencing facility (Dalhousie University, Halifax, Canada) using an Illumina MiSeq machine with a 2 × 300 bp chemistry, yielding sufficient high-quality sequences from all 73 Norwegian lake samples.

### DNA extraction and eukaryotic sequencing of Swedish lakes

DNA extraction was performed using polycarbonate filters using the PowerSoil DNA Isolation Kit (MO BIO, USA), following the method outlined by Juottonen and colleagues (23). For the preparation of eukaryotic libraries, a two-step PCR protocol was employed to minimize potential biases associated with PCR, such as chimera and heteroduplex formation (75). Additionally, this protocol allowed for the incorporation of barcodes, Illumina handles, and adapters into individual sample amplicons. In brief, approximately 1 ng of genomic DNA was added to duplicate PCR reactions containing dNTPs (0.2 mM), the eukaryotic primers 574*f (5′-ACACTCTTTCCCTACACGACGCTCTTCCGATCT-NNNN-CGGTAAYTCCAGCTCYV-3′) and 1132r (5′-AGACGTGTGCTCTTCCGATCT-CCGTCAATTHCTTYAART-3′) (76) at 0.25 µM, as well as Q5 High-Fidelity DNA Polymerase (New England Biolabs Inc; 0.4 unit) and PCR buffer (5×), in a final volume of 20 μL. The first PCR involved an initial denaturation step at 98°C for 1 min, followed by 20 cycles consisting of 10 s at 98°C, 30 s at 51°C, 1 min at 72°C, and a final elongation step of 2 min at 72°C. Duplicate amplicons were subsequently pooled and purified using the Agencourt AMPure XP purification system. A second PCR step was performed, utilizing the same PCR reagents/concentrations except for primers (detailed below). This step included an initial denaturation at 98°C for 30 s, followed by 12 cycles comprising 10 s at 98°C, 30 s at 66°C, and 72°C, with a final extension step of 2 min at 72°C. The previous PCR product (1 μL) served as the template for this second PCR, which introduced barcodes and complete Thruplex adapters for Illumina sequencing, with forward primer 5′-AATGATACGGCGACCACCGAGATCTACAC-[i5 index]-ACACTCTTTCCCTACACGACG-3′ and reverse primer 5′-CAAGCAGAAGACGGCATACGAGAT-[i7 index]-GTGACTGGAGTTCAGACGTGTGCTCTTCCGATCT-3′. Following a purification step with the Agencourt AMPure XP kit, quantification was conducted using a PicoGreen assay (Quant-iT PicoGreen, Invitrogen). Subsequently, SSU rRNA gene samples were pooled equimolarly, and sequencing was performed on a MiSeq instrument using 2 × 300 bp v3 chemistry and the software version 2.6.1.1 at the Science for Life Laboratory in Uppsala, resulting in high-quality sequencing data from a total of 83 Swedish lake samples.

### Fungus-specific sequencing

Fungal libraries were constructed employing a two-step PCR protocol with slight modifications. Genomic DNA ranging from 10 to 90 ng was added to duplicate PCR reactions containing dNTPs (0.2 mM), 0.4 units of Q5 polymerase, and PCR buffer (5×) in a final volume of 20 μL. Fungus-specific primers, including ITS3-Mix2 (5′-ACACTCTTTCCCTACACGACGCTCTTCCGATCT-NNNNNN-CAWCGATGAAGAACGCAG-3′) and an equimolar mixture of the reverse primers ITS4-cwmix1 (reverse; 5-GTGACTGGAGTTCAGACGTGTGCTCTTCCGATCT-NNNNNN-TCCTCCGCTTAYTGATATGC-3′) and ITS4-cwmix2 (reverse; 5′-GTGACTGGAGTTCAGACGTGTGCTCTTCCGATCT-NNNNNN-TCCTCCGCTTATTRATATGC-3′), were used. These primers were designed to include Illumina adapters and fungus-specific sequences described in references 16, 77. The resulting amplicons were approximately 350–500 bp in length, encompassing approximately 130 bp of the 5.8S rRNA gene and the entire ITS2 region, as previously proposed in references 13, 43.

The first PCR included an initial denaturation step of 98°C for 1 min, followed by 30 cycles of 20 s at 98°C, 30 s at 55°C, 1.5 min at 72°C, and a final extension of 7 min at 72°C. Duplicate amplicons were subsequently pooled and purified using the Agencourt AMPure XP purification system. The second PCR step was performed, utilizing 2 μL of the first purified PCR product as template and the same reagents/concentrations except for primers (identical to those used in the second PCR for SSU), including an initial denaturation step of 98°C for 30 s, followed by 15 cycles of 10 s at 98°C, 30 s at 66°C, 30 s at 72°C, and a final extension of 2 min at 72°C. The next steps, including PCR purification, quantification, and pooling, were performed as described for the SSU marker above. Illumina MiSeq 2 × 300 bp v3 sequencing was conducted at the Norwegian Sequencing Center from the University of Oslo, and the Science for Life Laboratory in Uppsala, for Norwegian and Swedish data sets, respectively. High-quality sequences were obtained from 61 Norwegian lake samples and 83 Swedish lakes.

### Bioinformatic analyses

Raw sequence data from the eukaryotic and fungal sequences were trimmed of primers with Cutadapt (78), and sequences without matching primers and fungal reads shorter than 200 bp were removed. Subsequently, each individual sequencing run (*n* = 4) was subjected to de-replication, denoising, and sequence pair assembly using the R package dada2 (79). Quality score plots were manually inspected, and for Norwegian eukaryotic amplicons, forward and reverse reads were trimmed to 270 and 190 bp length, respectively, while the Swedish eukaryotic amplicons were trimmed to 190 and 120 bp length, respectively. Fungus-specific sequences were not truncated at this step. Additional quality filtering eliminated sequences with unassigned base pairs and reads with a single Phred score below 20. After dereplication, forward and reverse error models were established in dada2, employing a subset of sequences (10^7^ bases for eukaryotic and 10^9^ bases for fungal forward and reverse reads) from each sequencing set. Eukaryotic forward and reverse reads were concatenated while fungal paired-end reads were merged. Chimeric sequences were removed using the “removeBiomeraDenova” function within the dada2 package, and amplicon sequence variants (ASVs) tables were obtained. Eukaryotic sequences were taxonomically assigned using the PR2 database (50).

Fungus-specific sequences underwent further processing: (i) extraction of the 5.8S rRNA gene and ITS2 region with ITSx (80) (ii—for ITS2) clustering of ASVs into OTUs at 97% similarity and removal of singletons using VSEARCH (81), (iii—for ITS2) curation of OTU tables with lulu (82) (iv—for 5.8S) dereplication and sorting using VSEARCH, (v—for both regions separately) taxonomic assignments of OTUs/ASVs against the UNITE database v. 8.3 (UNIT + INSD for eukaryotes) (83) using BLAST and lowest common ancestor analysis (LCA with parameters as follows: -b 8 -id 80 -cov 85 -t only_lca -flh unidentified), and finally (vi) merging the two taxonomic assignments to generate the final taxonomy using R.

### Data visualization and statistical analyses

All statistical analyses and data visualization were conducted using R version 4.2.1, with multiple R packages employed (for specific details, refer to the deposited R code). Missing values in the metadata were approximated using multiple imputations via the MICE algorithm (84), which employed variables as predictors that met specific criteria: (i) inclusion in the complete data model, (ii) relevance to non-response, (iii) explanation of a significant portion of variance, and (iv) absence of excessive missing values within the incomplete cases’ subgroup. In cases of autocorrelation, a subset of variables was retained for downstream analysis. PLS regression models were utilized to identify environmental variables predicting the contribution of fungal reads to the total read abundance in the eukaryotic data set (SSU 18S rRNA gene).

### Relationships between environmental properties and fungal community composition

Before conducting alpha and beta diversity analyses, eukaryotic and fungal data (ASV and OTU matrices, respectively) were rarefied to the smallest sample size within the respective eukaryotic (3,467 and 4,430 reads) and fungal (700 reads) data sets using the “rrarefy” function in the vegan package. Alpha diversity measures, including ACE and Pielou’s indices, were computed based on the rarefied community data. Beta diversity matrices were calculated using Bray–Curtis dissimilarity on the rarefied and Hellinger-transformed community data. Distance-based redundancy analysis was performed using the “capscale” function in vegan to identify environmental variables co-varying with fungal beta diversity. The significance of the dbRDA model was assessed through permutational analysis using the “anova.cca” function, both for the overall model and individual factors (marginal effects). Additionally, environmental vectors and factors were fitted onto Bray–Curtis ordinations (i.e., NMDS) to obtain correlation coefficients and significance levels with beta diversity.

### Incidence-based co-occurrence patterns and beta-diversity (β_RC_)

To assess stochastic versus deterministic community assembly processes across the two Scandinavian lake gradients, we employed incidence-based (presence/absence) EcoSimR (41). This approach utilizes Monte Carlo randomizations to create “pseudo-communities,” enabling statistical comparisons with the patterns in the actual data matrix. Various co-occurrence indices, including C-score (85), the number of checkerboard species pairs (47), and the number of species combinations (86), were estimated. Fixed rows-fixed columns and fixed rows-equiprobable columns simulations were utilized to minimize type i and type II errors (31). A higher C-score than expected by chance (C-score > 2 and *P* < 0.05) indicates low co-occurrence, whereas a lower C-score than expected by chance (C-score < −2 and *P* < 0.05) indicates high co-occurrence. Low co-occurrence can be interpreted as evidence for competitive exclusion (segregation), or differences in habitat preference. For the other co-occurrence indices (the number of checkerboard species pairs—“checkers,” and the number of species combinations—“combo”) results need to be interpreted the opposite way, with values greater than expected by chance suggesting competitive exclusion (segregation), or differences in habitat preference driving community assembly.

To validate the results from the co-occurrence analyses and determine whether communities assemble stochastically or deterministically, we calculated the incidence-based (Raup-Crick) dissimilarity indices (β_RC_). We used the “raup_crick” function provided in reference 33. β_RC_ values not significantly different from 0 indicate stochastic assembly, while values close to −1 suggest deterministic processes favoring similar communities and those close to +1 favor dissimilar communities. To evaluate differences in β_RC_ values among transient versus resident members of the lake communities, we operationally defined *Ascomycota* and *Basidiomycota* as transient, and *Chytridiomycota* and *Rozellomycota* as resident lake community members.

### Quantitative process estimates

To assess the relative contributions of potential species sorting, dispersal limitation, drift, and mass effects (referred to as “quantitative process estimates”), we adopted a two-step framework developed by Stegen et al. (28). This approach requires that phylogenetic distances (PD) among taxa should reflect differences in the ecological niches they occupy, thereby carrying a phylogenetic signal. We tested the presence of phylogenetic signals using Mantel correlograms (28). Furthermore, the prerequisite of this framework was met as the RDAs and the fitting of environmental vectors to ordination results with respect to phylogenetic community composition showed no correspondence.

To initiate the QPE based on pairwise comparisons (among sites during each sampling occasion), our first objective was to determine the extent to which the observed β_MNTD_ (β-mean-nearest-taxon-distance) deviated from the mean of the null distribution. To calculate the β_NTI_, we generated null communities using the standard tree-tip shuffling algorithm (“taxa.labels” with 1,000 permutations) to assess phylogenetic turnover against a random tree topology. When the observed β_MNTD_ significantly exceeded (β_NTI_ > 2) or fell below (β_NTI_ < −2) the null expectation, it indicated that the community assembly was driven by deterministic processes such as variable or homogeneous selection, respectively. In the second step, we then attributed |β_NTI_| < 2 by calculating the abundance-based beta-diversity using pairwise Bray-Curtis dissimilarity (β_RCbray_) (28). Communities that were not selected in the first step, thus not assembled by selection, were then characterized by deterministic processes such as (i) dispersal limitation coupled with drift if β_RCbray_  > +0.95, and (ii) homogenizing dispersal if β_RCbray_ <−0.95, or (iii) stochastic processes (ecological drift) if β_RCbray_ fell in between −0.95 and +0.95.

To further investigate whether “true” dispersal limitation might have played a role, we conducted Mantel correlation analyses between geographic distances (Euclidean distances of geographical coordinates) and fungal community dissimilarities (β_RCbray_) using Spearman correlation and 999 permutations. A significant relationship between β_RCbray_ and spatial distance would support the importance of dispersal limitation, while historical contingency would be deemed less significant. Moreover, we performed Mantel correlation analyses between β_RCbray_ and environmental dissimilarities (Euclidean distances) to explore whether community dissimilarities might be attributed to selection by environmental factors lacking a phylogenetic signal (“phylogenetically non-conserved selection”). Such factors might not have been detected in the first step but could be retained in the second step of the QPE analysis. To ensure that significant geographic distance effects were not confounded by spatially autocorrelated environmental variation, and vice versa, we conducted partial Mantel tests.

## Data Availability

The raw demultiplexed sequence data (eukaryotic and fungal amplicon reads) generated in this study have been deposited in the Sequence Read Archive (SRA) under BioProject accession code PRJNA1118258. The code used in this study is available at https://github.com/alper1976/fungal_diversity.

## SUPPLEMENTAL MATERIAL

Supplemental Material msystems.00691-26-s0001.docx Supplemental figures and tables.

ASM does not own the copyrights to Supplemental Material that may be linked to, or accessed through, an article. The authors have granted ASM a non-exclusive, world-wide license to publish the Supplemental Material files. Please contact the corresponding author directly for reuse.
