# Supplementary material for: Low determinism in community assembly of transient and resident pelagic fungi across climate zones in Scandinavian lakes
Source: mSystems. 2026 Aug 20;11(9):e00691-26. doi: 10.1128/msystems.00691-26 (PMC13595976; doi:10.1128/msystems.00691-26)
Supplement: Supplementary file 1 [file msystems.00691-26-s0001.docx]

**Supplementary material: Low determinism in community assembly of transient and resident pelagic fungi across climate zones in Scandinavian lakes**

Pedro M. Martin-Sanchez^1,2,*^, Christian Wurzbacher^3^, Laurent Fontaine^1,4^, Maria Jimenez Lara^1^, Heli Juottonen^5^, Nicolas Valiente^1,4,6^, Even Werner^1^, R. Henrik Nilsson^7^, Alexander Eiler^1,4*^

1 Section for Aquatic Biology and Toxicology, Department of Biosciences, University of Oslo, Blindernveien 31, 0371 Oslo, Norway

2 Environmental Microbiology and Cultural Heritage group, Instituto de Recursos Naturales y Agrobiología de Sevilla (IRNAS-CSIC), Avenida Reina Mercedes 10, 41012 Seville, Spain

3 Chair of Urban Water Systems Engineering, Technical University of Munich, Am Coulombwall 3, 85748 Garching, Germany

4 Centre for Biogeochemistry in the Anthropocene, Department of Biosciences, University of Oslo, Blindernveien 31, 0371 Oslo, Norway

5 Department of Biological and Environmental Science, University of Jyväskylä, P.O. Box 35, 40014 University of Jyväskylä, Finland

6 Department of Science and Agroforestry Technology and Genetics, University of Castilla-La Mancha, Campus Universitario s/n, 02071 Albacete, Spain

7 Department of Biology and Environmental Sciences, Gothenburg Global Biodiversity Centre, University of Gothenburg, Medicinaregatan 7B, Gothenburg, Sweden

^*^ Correspondence: pmartin@irnase.csic.es and alexander.eiler@ibv.uio.no

**Figure S1.** Map of the sampled Scandinavian lakes.

**Figure S2.** Spearman correlation between the environmental variables considered for the Norwegian (A) and Swedish (B) datasets.

**Figure S3.** Taxonomic composition of the eukaryotic phyla/kingdoms (following the PR^2^ taxonomy) in the Norwegian (A) and Swedish (D) datasets with the 15 most abundant divisions represented. Fungal sequences contributed 0.18% to 55% of the total eukaryotic reads as shown in the boxplot for the Norwegian (B) and Swedish (E) datasets. Relative abundances (percentages of reads) of the 15 most abundant divisions were inferred by redundancy analysis in relation to environmental parameters for the Norwegian (C) and Swedish (F) datasets.

**Figure S4.** Relative contribution of fungal operational taxonomic units (OTUs) to the overall community of each lake, categorized by their regional occurrence (unique to a single lake, present in 2–3 lakes, or found in >3 lakes) across the Norwegian (A) and Swedish (B) datasets.

**Figure S5.** The relative counts (or read numbers) of fungal OTUs in relation to their prevalence in the Norwegian (A) and Swedish (B) datasets.

**Figure S6.** Region-wide (A, Norway and B, Sweden) accumulation curves of fungal operational taxonomic units (OTUs).

**Figure S7.** Non-parametric multidimensional scaling plots based on Hellinger transformed fungal OTU matrices and Bray Curtis dissimilarities from the Norwegian (A) and Swedish (B) datasets. Dots represent individual lakes with color indicating their latitudinal position.

**Figure S8**. Taxonomic composition of the fungal genera (following the UNITE taxonomy) in the Norwegian (A) and Swedish (B) datasets with the 15 most abundant genera represented.

**Figure S9.** Venn diagram showing the overlaps of genera among the Norwegian and Swedish datasets.

**Figure S10.** Evaluation plots of generalized latent linear models for the fungal genera using the Norwegian lake dataset. Plots of Dunn-Smyth residuals, also known as randomized quantile residuals, are shown, including a plot of residuals against linear predictors of fitted values (A), a Normal Q-Q plot of residuals with a simulated point-wise 95% confidence interval envelope (B), residuals against row index (C) and column index (D) and scale-location plot (E).

**Figure S11.** Evaluation plots of generalized latent linear models for the fungal genera using the Swedish lake dataset. Plots of Dunn-Smyth residuals, also known as randomized quantile residuals, are shown, including a plot of residuals against linear predictors of fitted values (A), a Normal Q-Q plot of residuals with a simulated point-wise 95% confidence interval envelope (B), residuals against row index (C) and column index (D) and scale-location plot (E).

**Table S1.** Summary statistics of lake metadata included in the Norwegian and Swedish datasets.

**Table S2.** Results from partial Mantel tests and step-wise redundancy analyses. Partial Mantel tests were used to infer effect sizes of overall environmental and spatial factors on beta-diversity. By using a step forward selection model, we intended to select the variables most related to the Hellinger transformed Bray-Curtis dissimilarity matrix for the Norwegian (n = 61) and Swedish (n = 83) datasets using three levels of taxonomic resolution (i.e. operational taxonomic units - OTUs, genus and phylum).

**Table S3.** Results from partial Mantel tests and step-wise redundancy analyses. Partial Mantel tests were used to infer effect sizes of overall environmental and spatial factors on beta-diversity of the operationally defined transient and resident fungal community. By using a step forward selection model, we intended to select the variables most related to the Hellinger transformed Bray-Curtis dissimilarity matrix for the Norwegian and Swedish datasets using three levels of taxonomic resolution (i.e. operational taxonomic units - OTUs, genus and phylum).
